# Boost and Increased Antibody Breadth Following a Second Dose of PARVAX for SARS-CoV-2 in Mice and Nonhuman Primates

**DOI:** 10.3390/vaccines12080882

**Published:** 2024-08-02

**Authors:** Urja Bhatt, Cecile Herate, Reynette Estelien, Francis Relouzat, Nathalie Dereuddre-Bosquet, Dawid Maciorowski, Cheikh Diop, Emma Couto, Jillian Staiti, Mariangela Cavarelli, Laëtitia Bossevot, Quentin Sconosciuti, Page Bouchard, Roger Le Grand, Luk H. Vandenberghe, Nerea Zabaleta

**Affiliations:** 1 Grousbeck Gene Therapy Center, Ocular Genomics Institute and Schepens Eye Research Institute, Mass Eye and Ear, Boston, MA 02114, USAluk_vandenberghe@meei.harvard.edu (L.H.V.); 2 Department of Ophthalmology, Harvard Medical School, Boston, MA 02114, USA; 3 The Broad Institute of Harvard and MIT, Cambridge, MA 02142, USA; 4 Harvard Stem Cell Institute, Harvard University, Cambridge, MA 02138, USA; 5 Center for Immunology of Viral, Auto-Immune, Hematological and Bacterial Diseases (IMVA-HB/IDMIT), Université Paris-Saclay, Inserm, CEA, 92260 Fontenay-aux-Roses, France; 6 Ciendias Bio, Weston, MA 02493, USA

**Keywords:** PARVAX platform, genetic vaccine, re-administration, boost, SARS-CoV-2, antibody breadth, anti-vector response

## Abstract

PARVAX is a genetic vaccine platform based on an adeno-associated vector that has demonstrated to elicit potent, durable, and protective immunity in nonhuman primates (NHPs) after a single dose. Here, we assessed vaccine immunogenicity following a PARVAX prime-boost regimen against SARS-CoV-2. In mice, a low-dose prime followed by a higher-dose boost elicited potent neutralizing antibody responses and distinct cross-reactivity profiles, depending on the antigen used in the booster vaccine. However, the potent neutralizing anti-vector antibody responses developed in mice limited the dose that could be administered as a prime. We further explored the re-administration efficacy in NHPs primed with a SARS-CoV-2 Delta vaccine and boosted with an Omicron BA.1 vaccine at week 15, after the primary response peak antibody levels were reached. The boost elicited an increase in antibodies against several Omicron variants, but no increase was detected in the antibody titers for other variants. The anti-vector responses were low and showed some increased subsequent boosts but generally declined over time. The potent prime vaccination limited the detection of the boosting effect, and therefore, the effect of anti-vector immunity was not fully elucidated. These data show that PARVAX can be effectively re-administered and induce a novel antigenic response.

## 1. Introduction

The public health emergency caused by COVID-19 triggered an unprecedented volume of research to test various vaccine platforms against the virus (https://www.who.int/publications/m/item/draft-landscape-of-covid-19-candidate-vaccines (accessed on 1 February 2024)) [1,2,3,4]. Among these, we developed the PARVAX platform, a genetic vaccine based on adeno-associated virus (AAV) [5,6]. This platform uses a specific AAV serogroup of capsids, which includes rh32.33 and AAV11, with minimal natural pre-existing immunity in humans [6,7]. Importantly, unlike other vectors in the AAV class, these serotypes exhibit a tropism for antigen-presenting cells (APCs) and activate the toll-like receptor (TLR) 9 pathway, thereby effectuating a cytotoxic T-cell response toward the encoded transgene antigen that results in the elimination of transduced cells, thus limiting vector persistence and expression [8,9,10]. PARVAX candidates for SARS-CoV-2 demonstrate durable, up to 2-year neutralizing antibody responses, which provide near-sterilizing upper and lower airway protection in cynomolgus macaques after a single-dose administration. These qualities combined with established commercial and low-cost manufacturing processes and room temperature stability make this platform imminently feasible, scalable, and affordable for vaccine applications [6].

Viral vector-based vaccines, such as adenovirus vaccines, are genetic vaccines that consist of a genetically engineered virus that carries the gene of the antigen of interest. Upon the administration of the vaccine, the antigen is expressed in resident cells, and the transduction of the viral vector acts as adjuvant to the vaccination [8,9,10]. Some of these viral vector platforms have demonstrated the potential to induce potent humoral and cellular responses against the antigen, even following a single dose in certain cases [11,12]. However, some viral vectors are based on viruses that circulate in human populations, and consequently, their utility may be limited in individuals who have anti-vector antibodies due to a prior natural infection [13]. While many AAVs have significant pre-existing immunity in humans, PARVAX’s AAV technology is minimally seroprevalent [7]. Regardless of the anti-vector immunity level caused by natural infection, any administration in the context of a vaccine or gene therapy application with a serologically cross-reactive viral vector may hamper future administrations of the same modality [14]. In the context of PARVAX, prior data indicate minimal to no cross-reactivity of the particular AAV serotype used with commonly used gene therapy vectors in nonhuman primate studies (as well as in previous studies in rabbits) [7].

Here, we sought to assess whether the PARVAX platform can provide a boost following a second administration in mouse and nonhuman primate models. While PARVAX was shown to lead to sustained antibody responses from a single-dose administration, a second administration may be desired to complement breadth or extend the duration of the primary response (e.g., to address VOCs in COVID-19) or as a vaccine for another class of immunogens (e.g., influenza following COVID-19).

## 2. Material and Methods

### 2.1. Vaccine Candidates

AC3 is an adeno-associated virus serotype rh32.33 (AAVrh32.33)-based candidate that carries a secreted monomeric S1 domain of Wuhan SARS-CoV-2 Spike antigen.

ACM1, ACM-Delta, ACM-BA.1, and ACM-BA.2 are adeno-associated virus serotype 11 (AAV11)-based candidates that carry the codon optimized, pre-fusion stabilized (furin cleavage site mutated to G682SAS685 and P986P987 substitutions) full-length Wuhan, Delta, BA.1, and BA.2 VOC Spike transgene under the control of a minimal cytomegalovirus (CMV) promoter (deposited to GenBank). ACM-SARS1 carries the same version of the SARS-CoV Spike antigen.

The candidates were produced at the Mass Eye and Ear/Schepens Eye Research Institute Gene Transfer Vector Core (https://www.vdb-lab.org/vector-core/ (accessed on 21 July 2024)) using a previously described protocol [6]. Briefly, vaccine candidates were produced in HEK293 cells by triple transfection using polyethylenimine or PEI Max (Polysciences, Cat #24765-2-, Polysciences, Inc., Warrington, PA, USA) of pACM-Delta ITR-flanked transgene, pKan2/11 (AAV2 rep and AAV11 capsid construct), and pALD-X80 adenoviral helper plasmid at a 1:1:2 ratio. Cells were seeded in 10-layer HYPERFlasks, and the PEI-Max/DNA ratio was 1.375:1 (*v*/*w*). Vectors were harvested 72 h after transfection using Benzonase (EMD Millipore, catalog no. 1016970010, Sigma Aldrich, Dorset, UK) to degrade DNA/RNA. Vectors were concentrated by tangential flow filtration and purified by iodixanol gradient ultracentrifugation [15]. The concentration of the vaccine candidates was assessed by ddPCR according to a previously published protocol [16].

### 2.2. Mice

Mouse studies and protocols were approved by the Schepens Eye Research Institute IACUC. Six–eight-week-old C57BL/6 or BALB/c mice were injected intramuscularly (IM) in the right gastrocnemius (volume: 25 μL) with different doses of vaccine candidates on days 0 and 28. The re-administration was performed in the same muscle. Animals received the following vaccine candidates and doses in the different studies: AC3 candidate at 10^9^ genome copies (gc), 10^10^ gc, or 10^11^ gc; ACM1 at 2 × 10^9^ gc or 10^11^ gc; ACM-BA.2 at 10^11^ gc; ACM-SARS1 at 10^11^ gc and a mixture of ACM-BA.2 and ACM-SARS1 at a total dose of 10^11^ gc. Blood was harvested by submandibular bleeds, and serum was isolated.

### 2.3. Nonhuman Primates (NHPs)

Cynomolgus macaques (*Macaca fascicularis*), aged 37–54 months at a first immunization (1 female and 7 males) and originating from Mauritian AAALAC-certified breeding centers were housed in IDMIT facilities (CEA, Fontenay-aux-Roses, France) (Animal facility authorization #D92-032-02, Préfecture des Hauts de Seine, France) and in compliance with European Directive 2010/63/EU, the French regulations, and the Standards for Human Care and Use of Laboratory Animals of the Office for Laboratory Animal Welfare (OLAW; assurance number # F22-00556, US). The protocols were approved by the institutional ethical committee “Comité d’Ethique en Expérimentation Animale du Commissariat à l’Energie Atomique et aux Energies Alternatives” (CEtEA #44) under the statement number A20-061. The study was authorized by the “Research, Innovation and Education Ministry” under the registration number APAFIS#28946-2021011312169043 v2.

Animals were vaccinated with 10^11^ genome copies (gc) via intramuscular injection of the ACM-Delta vaccine candidate on week 0. On week 15, all animals were re-administered, 4 animals were re-administered with 10^11^ gc of the ACM-BA.1 vaccine candidate, and 4 animals were re-administered with a saline solution containing PBS, NaCl, and a small amount of Pluronic. Blood samples were harvested on weeks 0, 2, 4, 8, 12, 15, 17, 19, 24, 28, 32, and 48.

### 2.4. Quantification of SARS-CoV-2 IgG Antibodies

Cynomolgus macaque sera were screened for spike-specific IgG against SARS-CoV-2 wild-type and variants Alpha, Beta, Delta, and Omicron subvariants BA.1, BA.2, and BA.3 using the V-PLEX SARS-CoV-2 Panel 25 IgG kit (MesoScale Discovery (MSD), Rockville, MD, USA) according to the manufacturer’s instructions and as previously described [17]. Briefly, a 30 min room-temperature blocking step was performed using 50 μL of blocker A (1% BSA in MilliQ water) shaking at 700 rpm in a digital microplate shaker. Samples were diluted at ratios of 1:500 and 1:5000 in a diluent buffer and added in duplicates (50 μL/well). A standard curve and a blank well were included as controls. After the samples were incubated for 2 h at room temperature and shaken, the plates were washed three times with 150 μL of the MSD kit Wash Buffer. Fifty milliliters of the secondary SULFO-tagged anti-Human IgG antibody was added and incubated under the same conditions for 1 h. After three washes, 150 μL of MSD GOLD Read Buffer B was added to each well. The plates were read immediately after on a MESO QuickPlex SQ 120 machine (MSD, Rockville, MD, USA). Electro-chemioluminescence (ECL) signals were recorded, and the results were expressed as AU/mL.

### 2.5. SARS-CoV-2 and AAV-Binding Antibody ELISA

ELISA 96-well plates (Thermo Fisher Scientific; Cat# 44-2404-21, Thermo Fisher Scientific, Waltham, MA, USA) were coated overnight at 4 °C with 1 µg/mL SARS-CoV-2 RBD or AAV11 particles diluted in phosphate-buffered saline (PBS). Biotek 405 TS Microplate washer (BioTek Instruments, Inc., Winooski, VT, USA) was used for all the washes, consisting of five washes with 200 μL of PBS-Tween 20 0.05% (Sigma; Cat# P2287-100ML, Sigma Aldrich, Dorset, UK). Plates were washed and blocked for 2 h at room temperature with 200 µL of Blocker Casein in PBS (Thermo Fisher Scientific; Cat# 37528, Thermo Fisher Scientific, Waltham, MA, USA). Serial dilutions of serum samples starting at a 1:100 dilution were added to the plates (100 µL/well) and incubated for 1 h at room temperature. After washing, 100 µL of secondary rabbit Anti-Monkey IgG (whole molecule)-Peroxidase antibody (Sigma-Aldrich; Cat# A2054, RRID:AB_257967, Sigma Aldrich, Dorset, UK) diluted at a ratio of 1:10,000 or anti-Monkey IgA (NIH Nonhuman Primate Reagent Resource supported by AI126683 and OD 010976) diluted at a ratio of 1:5000 was added and incubated for 1 h. Lastly, the plates were developed using 100 µL of Seracare SureBlue Reserve^TM^ TMB Microwell Peroxidase Substrate solution (SeraCare, Cat# 53-00-03, SeraCare, Milford, MA, USA). One hundred milliliters of Seracare KPL TMB Stop Solution (SeraCare, Cat# 50-85-06, SeraCare, Milford, MA, USA) was added to each well to stop the reaction. Optical density (OD) at 450 nm was measured using a Biotek Synergy H1 plate reader (BioTek Instruments, Inc., Winooski, VT, USA). The titer was the reciprocal of the highest dilution with absorbance values higher than four times the average of the negative control wells.

### 2.6. Pseudovirus Neutralizing Assay

This assay was performed as previously described [6]. Briefly, HEK293T cells were used to produce pseudoviruses by triple transfection of psPAX2, pCMV-SARS2-Spike (WT or VOC), and pCMV-Lenti-Luc. Supernatants were harvest 2 days later, centrifuged and filtered to remove cell debris. The TCID50 was calculated by limiting dilution in ACE2-overexpressing HEK293T cells. Once the pseudoviruses were ready, the neutralization assay was performed in HEK293T-ACE2 cells. Serum samples were heat-inactivated and serially diluted. The samples were incubated with the different pseudoviruses for 45 min at 37 °C before adding them to the HEK293T-ACE2 cells. Two days later, the luciferase signals were quantified in the HEK293T-ACE2 cells to calculate the EC50 values for each serum sample.

### 2.7. AAV Neutralization Assay

2 × 10^4^ per well HEK293 cells were seeded in poly-L-lysine pre-coated 96-well black plates. The next day, heat-inactivated serum samples were serially diluted in DMEM and incubated for 1 h with an MOI of 10^4^ of AAVrh32.33-expressing luciferase. Subsequently, the medium was removed from the HEK293 cells, and 50 µL of the serum-AAV mix was added. After an incubation of 1 h at 37 °C, complete 150 µL DMEM media (with fetal bovine serum) were added. Forty-eight hours later, luciferase signals were measured to calculate the neutralization titers for each serum sample.

### 2.8. IFN-γ ELISPOT Assay in Peripheral Blood Mononuclear Cells (PBMCs)

IFNγ ELISpot assay was performed in cynomolgus macaque PBMCs using the Monkey IFNg ELISpot PRO kit (Mabtech, #3421M-2APT, Mabtech Inc., Cincinnati, OH, USA) according to the manufacturer’s instructions. Briefly, 200,000 PBMCs/well were plated and stimulated with BA.1, Wuhan, or Delta SARS-CoV-2 spike peptides (PepMixTM) synthetized by JPT Peptide Technologies (Berlin, Germany). The peptides were 15 amino acids long with 11 amino acid overlaps and prepared at a concentration of 2 µg/mL. After 18 h of incubation at 37 °C, cells were washed 5 times with PBS before a 2 h incubation at 37 °C with a biotinylated anti-IFNγ antibody. After 5 washes, spots were developed by adding a BCIP/NBT-plus substrate solution and counted with an automated ELISpot reader ELRIFL04 (Autoimmun Diagnostika GmbH, Strassberg, Germany). Spot-forming cells (SFCs) per 10^6^ PBMCs were calculated by averaging duplicate wells.

### 2.9. Statistical Analysis

GraphPad Prism 9 was used for graphical representation and statistical analyses. Mann−Whitney test or Kruskal−Wallis test and Dunn’s test were used to compare groups. The Wilcoxon matched-pairs signed-rank test (dependent samples, *n* < 10) was used to compare pre- and post-boost measurements. 

## 3. Results

### 3.1. High Prime Doses of PARVAX Eliciting High Anti-Vector and Anti-Antigen Responses in Mice That Impede Boosts

**Figure 1:**
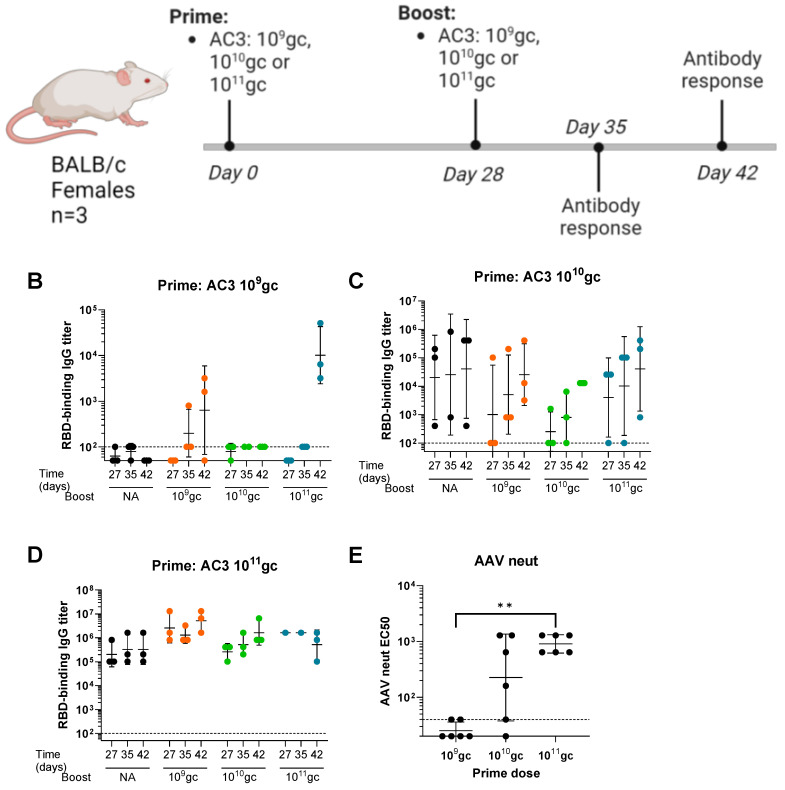
Feasibility of the 1st-generation PARVAX re-administration at different prime doses in mice. (**A**) Study design. (**B**–**D**) Wuhan RBD-binding antibody titers in animals primed with different doses of the AC3 vaccine (10^9^ gc (**B**), 10^10^ gc (**C**), or 10^11^ gc (**D**)) and boosted on day 28 with different boost doses (black circles: single dose; orange circles: 10^9^ gc boost; green circles: 10^10^ gc boost; blue circles: 10^11^ gc boost). (**E**) Anti-AAVrh32.33-neutralizing antibodies on day 27 (before the boost) in animals primed with different doses of the AC3 vaccine candidate. Kruskal−Wallis and Dunn’s post-test; ** *p* < 0.01.

A preliminary study was performed to assess the potential of PARVAX for a successful re-administration in mice. BALB/c female animals received one or two vaccinations (day 0 and day 28) of an early PARVAX candidate (AC3), which expressed a soluble monomeric form of the S1 domain of SARS-CoV-2 Spike [6]. Different doses (10^9^ gc, 10^10^ gc, and 10^11^ gc) and all possible prime-boost combinations were tested (Figure 1A). Animals receiving a single dose on day 0 were included as controls. Spike receptor-binding-domain (RBD)-binding antibodies were measured before (day 27) and after the boost (days 35 and 42). Animals primed with 10^9^ gc did not develop antibodies before the boost, and the group boosted with 10^11^ gc developed antibodies following the kinetics expected after a high dose (Figure 1B). The 10^10^ gc dose as the prime showed high variability at pre-boost anti-RBD antibody levels after the prime, which hampered the assessment of the effect of the second dose (Figure 1C). The group primed with 10^11^ gc was not successfully boosted with any of the doses (Figure 1D). Anti-vector antibody responses revealed high levels of neutralizing antibodies in the animals primed with the 10^11^ gc dose at day 28 before the boost, which likely explained the absence of boosting effect (Figure 1E). Additionally, the highest dose elicited high anti-antigen responses, possibly reaching a plateau and impeding a further boost. The lowest 10^9^ gc dose did not elicit anti-vector responses (above the limit of sensitivity tested), and it also failed to elicit anti-spike responses. Finally, the mid-dose induced variable anti-vector and anti-spike responses that did not allow assessing the effect of the boost.

### 3.2. Potent Boosting Effects Observed When Using Low Doses of Optimized PARVAX Candidates as the Prime

Considering the previous results, we hypothesized that a more potent PARVAX candidate may achieve immunogenicity in a more consistently robust manner at a lower dose. These features may allow for a second PARVAX vaccine application to be effective and measurable at the lower dose. As previously reported, increased PARVAX potency was achieved largely by incorporation of a stronger promoter to drive antigen expression and confirmed to yield potent and protective immunogenicity in vivo. The optimized vaccine contains a potent minimal CMV promoter that enables a 10-fold higher expression of the antigen and elicits protective immunity at 10-fold lower doses [5].

**Figure 2:**
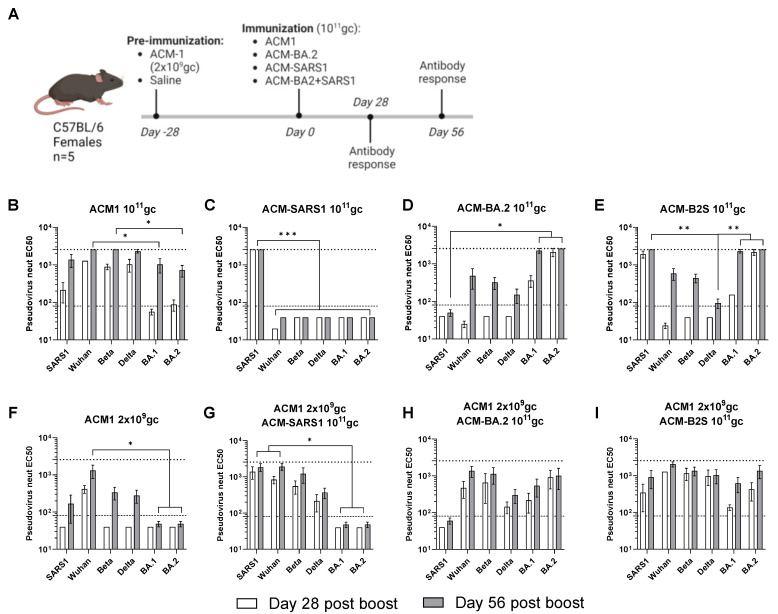
Boosts with VOC candidates following a low-dose prime of the 2nd-generation PARVAX. (**A**) Study design. (**B**–**E**) Neutralizing responses against different variants of concerns in naïve animals vaccinated with 10^11^ gc of PARVAX candidates for Wuhan (ACM1) (**B**), SARS1-CoV-1 (ACM-SARS1) (**C**), BA.2 (ACM-BA.2) (**D**), or a bivalent ACM-SARS1 and ACM-BA.2 candidate (ACM-B2S) (**E**). (**F**–**I**) Neutralizing responses against different variants of concerns in animals pre-immunized with 2 × 10^9^ gc of ACM1 and boosted with saline (**F**) or PARVAX candidates for SARS1-CoV-1 (ACM-SARS1) (**G**), BA.2 (ACM-BA.2) (**H**), or a bivalent ACM-SARS1 and ACM-BA.2 candidate (ACM-B2S) (**I**). (**B**–**I**) Kruskal−Wallis test and Dunn’s post-test in day-56 post-boost data. * *p* < 0.05, ** *p* < 0.01, and *** *p* < 0.001.

Here, C57BL/6 animals (n = 5) were vaccinated on day 0 of the study with a high total dose of 10^11^ gc of PARVAX candidates that expressed different antigens: BA.2 VOC Spike (ACM-BA.2), SARS-CoV1 Spike (ACM-SARS1), and a combination of both (ACM-B2S) (Figure 2A). Half of the animals were pre-immunized with a low dose of 2 × 10^9^ gc of the optimized vaccine (ACM1) that expressed the full-length prefusion-stabilized Wuhan Spike and was previously shown to elicit anti-spike antibody responses. Neutralizing antibodies against several spikes (SARS1, Wuhan, Beta, Delta, BA.1, and BA.2) were measured on days 28 and 56 of the study. Figure 2B–E shows the neutralization patterns in animals that received a single shot of 10^11^ gc of the PARVAX candidates on day 0. Vaccinations with different VOCs were included in this study to analyze if a boost with a different variant can trigger improved breadth. The ACM1 candidate that expressed the Wuhan Spike antigen provided a relatively broad neutralization profile of the tested variants, with significantly lower levels of anti-BA.1- and anti-BA.2-neutralizing antibodies (Figure 2B). The SARS1 and BA.2 candidates individually elicited potent homologous neutralization and much less or no response against other VOCs (Figure 2C,D). The combined vaccine showed an additive effect (Figure 2E). This cross-reactivity profiles can be explained by the degree of antigenic similarity of the different variants, in which the SARS1 and BA.2 variants are more antigenically distinct from the other tested variants and also between each other as shown by several antigenic cartography studies [18,19]. Figure 2F–I shows the neutralization profiles in animals that were pre-immunized with a low dose of ACM1. A single-low-dose vaccination elicited neutralization of the Wuhan Spike but limited cross-reactivity to VOCs tested (Figure 2F), in comparison to ACM1 at high-dose in Figure 2B, illustrating the dose effect on cross-reactivity of the PARVAX vaccine response. The pre-immunized animals that received a second PARVAX dose with different VOC antigens presented more potent responses than the primed-only animals (Figure 2F vs. Figure 2G–I), indicative of the biological activity (and thus lack of neutralization) of the second PARVAX dose. Additionally, the breadth of antibody responses was enhanced in the prime/boosted animals compared to in the naïve immunized animals (Figure 2C vs. Figure 2G; Figure 2D vs. Figure 2H; Figure 2E vs. Figure 2I). For example, the cross-reactivity in the animals that received ACM-SARS1 after a low dose of ACM1 (Figure 2G) appeared stronger, broader and had faster kinetics than the sum of its parts (Figure 2C,F). Finally, the more antigenically distinct SARS-CoV spikes included [18,19], the broader the neutralizing response was. This is demonstrated in Figure 2I where animals receiving Wuhan spike as prime and a combined BA.2 and SARS1 spikes (antigenically distinct spikes) as boost elicit equally high neutralization to all tested variants. These results show that PARVAX can be successfully re-administered with low anti-vector pre-existing immunity and that heterologous boosts elicited different antibody cross-reactivity profiles.

### 3.3. Evidence of Effective PARVAX Re-Administration in Nonhuman Primates

Next, we evaluated the re-administration of PARVAX in larger animals, specifically in cynomolgus macaques. In previous NHP studies, the anti-AAV-neutralizing antibody level elicited by PARVAX at a high dose of 10^12^ gc per animal was low [6]. We hypothesized that in large animals, with the 2nd-generation PARVAX vaccine being more potent, a 10-fold lower vaccine dose would elicit minimal anti-AAV immunogenicity and allow for a second administration to demonstrate its effectiveness.

**Figure 3:**
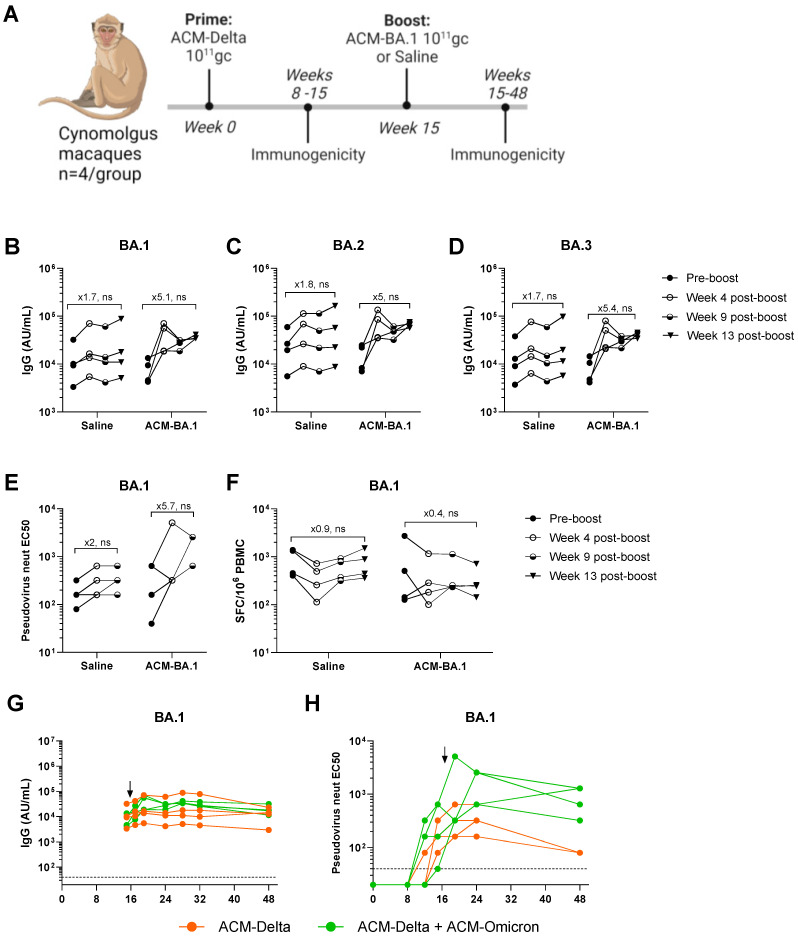
Re-administration of the 2nd-generation PARVAX in nonhuman primates. (**A**) Study design. (**B**–**D**) Binding antibody responses (AU/mL) in boosted and non-boosted animals against BA.1 (**B**), BA.2 (**C**), and BA.3 (**D**) on week 15 (pre-boost), week 19 (4 weeks post-boost), week 24 (9 weeks post-boost), and week 28 (13 weeks post-boost). (**E**) BA.1-neutralizing antibody responses on week 15 (pre-boost), week 19 (4 weeks post-boost) and week 24 (9 weeks post-boost). (**F**) IFN-gamma spot-forming cells (SFCs) after BA.1 Spike protein peptide stimulation of peripheral blood mononuclear cells (PBMCs) extracted on week 15 (pre-boost), week 19 (4 weeks post-boost), week 24 (9 weeks post-boost), and week 28 (13 weeks post-boost). (**G**,**H**) Longitudinal anti-BA.1-binding antibody (AU/mL) (**G**) and -neutralizing antibody (**H**) responses up to week 48 after prime administration with arrows indicating timing of second PARVAX administration (ACM-BA.1 or Saline). (**B**–**F**) Wilcoxon matched-pairs signed-rank test (no significant (ns) in all cases).

**Figure 4:**
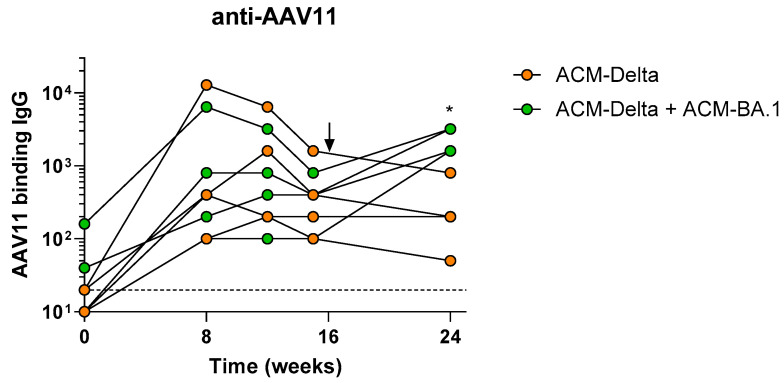
Anti-vector antibody responses in nonhuman primates. Anti-AAV11-binding antibodies in animals vaccinated once or twice with ACM candidates with arrow indicating timing of second administration of ACM.BA-1 (green) or Saline (orange). Mann−Whitney test comparison between groups at each timepoint. * *p* < 0.05.

We, therefore, vaccinated eight NHPs with the Delta VOC PARVAX candidate (ACM-Delta) with 10^11^ gc per animal, a dose previously shown to protect them from a live-viral challenge [5]. On week 15, after the antibody peak was reached, four of the animals were boosted with 10^11^ gc of Omicron BA.1 PARVAX (ACM-BA.1) alongside four animals that received saline as controls. Immunogenicity was monitored regularly during the 48 weeks of the study (Figure 3A). The boosted animals showed an increase of ~fivefold in binding antibodies against the VOCs BA.1, BA.2, and BA.3 of the Omicron family (Figure 3B–D), confirming the effectiveness of the booster vaccine on novel antigens. A similar increase in pseudovirus-neutralizing antibodies was observed in the boosted animals (Figure 3E). Binding and neutralizing antibodies against other VOCs, including the Delta antigen included in the prime vaccine, did not show a distinct response in the boosted compared to in non-boosted animals (Supplementary Figure S1). The antibody responses remained stable at peak levels through the last measurement at week 48 (Figure 3G,H and Figure S2). The cellular immune response as measured by IFN-γ ELISPOT was not different in the boosted compared to in the non-boosted animals (Figure 3F and Figure S3). Finally, we measured the anti-vector immune response before and after the boost (Figure 4). We found that low levels of AAV11-binding antibodies were generated in all animals after the prime dose which tended towards reduction in the animals boosted with saline while animals boosted with the vector showed a significant increase in antibodies after the boost dose (Figure 4).

## 4. Discussion and Conclusions

The COVID-19 pandemic highlights the importance of developing and characterizing novel vaccine platforms prior to the emergence of the next pandemic pathogen. Over the past years, the world has witnessed the expedited development and approval of several efficacious and safe gene-based vaccines for COVID-19, such as mRNA- and adenovirus-based vaccines, which saved millions of lives [20]. These new technologies, when deployed to a large population, have shown to be efficacious at preventing severe diseases [21]. Several features of the SARS-CoV-2 pandemic, including the length of the pandemic threat and the emergence of partially immune-escape VOCs, require repeat immunization of the population at certain intervals with updated vaccines to address both the waning of vaccine immunogenicity and the adjustment of the vaccine to the newly emerged circulating strains [22].

Previously, we have shown that PARVAX vaccine candidates for COVID-19 elicit potent and durable antibody responses following a single dose, detecting sustained peak antibody levels for up to 2 years in NHPs, which resulted in strong protection upon challenge with several SARS-CoV-2 VOCs in both the lower and the upper respiratory tracts. We also reported that this platform is stable at room temperature for up to 1 month and that it is amenable to large-scale manufacturing in a commercially validated process [5,6]. All these features make PARVAX an attractive platform for the development of vaccines against existing and future pathogens.

However, as PARVAX is a viral vector vaccine technology that has the potential to induce a neutralizing anti-vector immune response, an important question as to whether it can be effective in a second administration, e.g., as a homologous or heterologous boost for COVID-19 VOCs or for utility for subsequent use of multiple vaccine antigens (e.g., SARS-CoV-2 followed by influenza). In the broader AAV literature, neutralizing anti-vector responses are well established particularly in the following contexts: (a) primary administrations at high doses that are common in gene therapy applications and (b) of secondary systemic administrations such as intravenous liver-directed gene therapy applications [23,24]. There is some evidence that neutralizing AAV antibodies in NHPs can be overcome to allow for effective AAV administration when given via the intramuscular route of injection [25]. We hypothesize that the microenvironment of muscle parenchyma limits the opportunity for an IM-injected AAV product to encounter neutralizing antibodies prior to reaching its cellular transduction target, particularly at low systemic serology NAB titers. 

In support of this hypothesis is the adenoviral vaccine experience, now widely deployed as vaccines for COVID-19 and previously also approved for Ebola [4,26,27,28]. These vector systems face the same concerns of development of anti-vector antibodies upon vaccination as PARVAX does. The effect of anti-vector immunity upon vaccination has been studied, and it has been reported that anti-vector immunity upon vaccination does not influence the vaccination significantly in the context of pre-existing immunity or re-administration [12,13,14,28].

The present study shows that PARVAX can be re-administered effectively but requires that the primary vaccine be potent at a sufficiently low dose that limits anti-AAV-neutralizing antibody induction. This proves to be easier to achieve in larger animal models such as NHPs (and perhaps by extension in humans) compared to in mice in our studies. We hypothesized that this is due to the following reasons: (1) a higher relative potency of PARVAX in NHPs (e.g., the same absolute 10^11^ gc dose achieves similar neutralizing titers to encoded antigens mice and NHPs, notwithstanding a greater than 100-fold difference in body weight) [5,6], (2) a reduced impact of circulating anti-AAV-neutralizing antibodies with a small volume local administration in the larger muscle mass of the NHP [25], and (3) the relatively lower induction of anti-AAV-neutralizing antibodies of a low-dose AAV in NHPs compared to in mice [5,6].

Indeed, mouse studies show that very high anti-vector antibody levels are elicited even with modest AAV IM doses, and these responses prevent effective re-administration. Only when lower non-immunogenic prime doses (e.g., 2 × 10^9^ gc) are used, can effective boosting be achieved. In the present study, NHPs primed with a PARVAX candidate for Delta VOC and boosted with a candidate for BA.1 showed an increase in the antibody titers against the VOCs in the Omicron family (BA.1, BA.2, and BA.3) relative to animals that only received the prime even in presence of moderate anti-vector responses.

Further studies are needed to understand the lack of the boost response observed for VOC antigens included in the prime when boosted with a heterologous vaccine dose. This may be due to the fact that, given the durability and potency of the PARVAX prime, responses are already so high that a boost response is limited or harder to detect. In addition, while these studies illustrate that a second administration is feasible for the PARVAX application in COVID-19, a more careful quantitative characterization on the relationship between the dose and anti-AAV neutralizing antibody response in the context of an IM-injected PARVAX vaccine is needed. Additionally, the PARVAX platform may face further limitations if a 3rd or 4th administration is required to adapt the vaccine to other variants or other pathogens. The antibody levels could further increase upon subsequent administrations, and neutralization of the vector may lead to decreased vaccine efficacy. This means that the durability elicited by PARVAX may permit longer intervals between vaccinations, which will enable reduction of anti-vector antibody responses and improvement of the efficacy of subsequent vaccinations. All of these hypotheses and challenges need to be explored in further studies.

## Data Availability

The original contributions presented in the study are included in the article and Supplementary Materials. Further inquiries can be directed to the corresponding author.

## Supplementary Materials

The following supporting information can be downloaded at: https://www.mdpi.com/article/10.3390/vaccines12080882/s1, Three figures (Figures S1–S3) are included in Supplementary Materials. Figure S1: Binding and neutralizing antibodies to VOCs in PARVAX boosted and not boosted non-human primates; Figure S2: Longitudinal antibody responses in PARVAX boosted and not boosted non-human primates; Figure S3: Cellular immune responses in PARVAX boosted and not boosted non-human primates.
