# Supplementary material for: Boost and Increased Antibody Breadth Following a Second Dose of PARVAX for SARS-CoV-2 in Mice and Nonhuman Primates
Source: Vaccines (Basel). 2024 Aug 2;12(8):882. doi: 10.3390/vaccines12080882 (PMC11359472; doi:10.3390/vaccines12080882)
Supplement: Supplementary file 1 [file vaccines-12-00882-s001.zip › vaccines-3092462-supplementary.pdf]

Supplemental Material

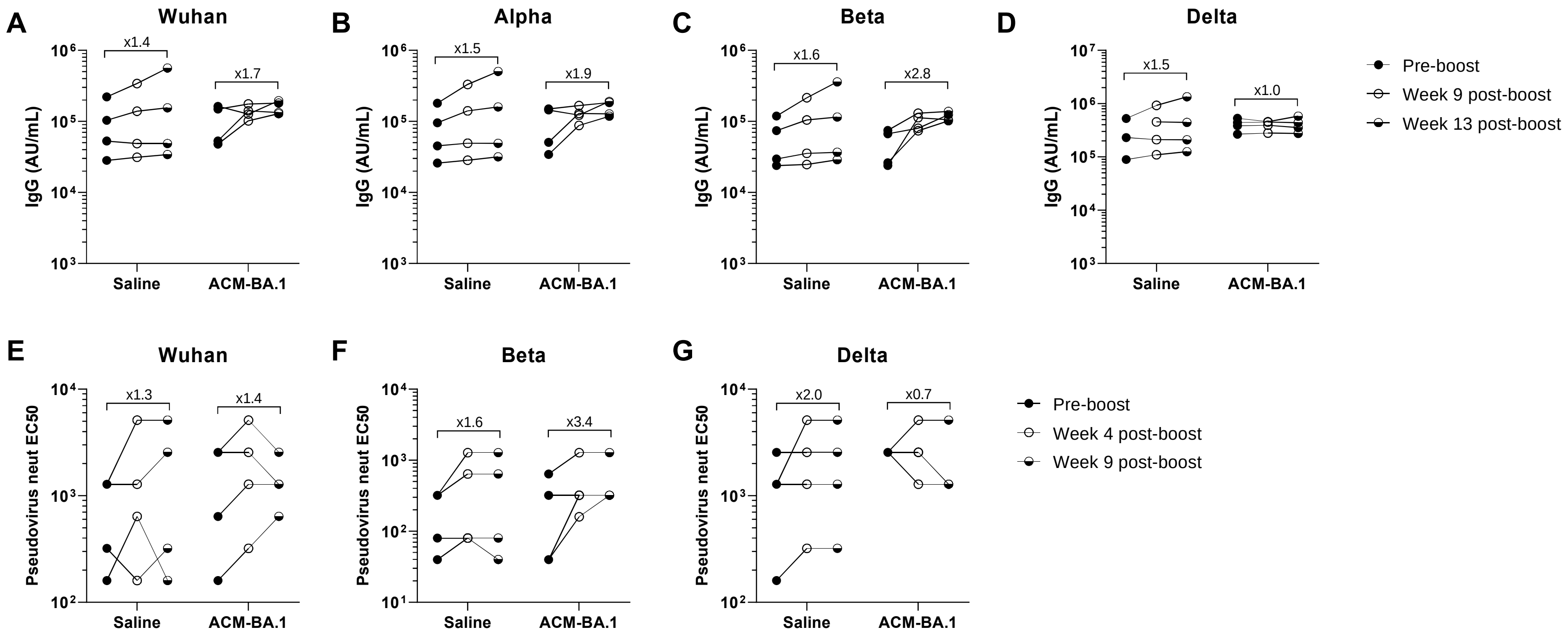

Supplemental Figure S1. Binding and neutralizing antibodies to VOCs in PARVAX boosted and not boosted non-human primates. **A-D)** Binding antibody responses (AU/mL) in boosted and not boosted animals against Wuhan (A), Alpha (B), Beta (C) and Delta (D) on week 15 (pre-boost), week 24 (9 weeks post-boost) and week 28 (13 weeks post-boost). **E-G)** Wuhan (E), Beta (F) and Delta (G) neutralizing antibody responses on week 15 (pre-boost), week 19 (4 weeks post-boost) and week 24 (9 weeks post-boost).

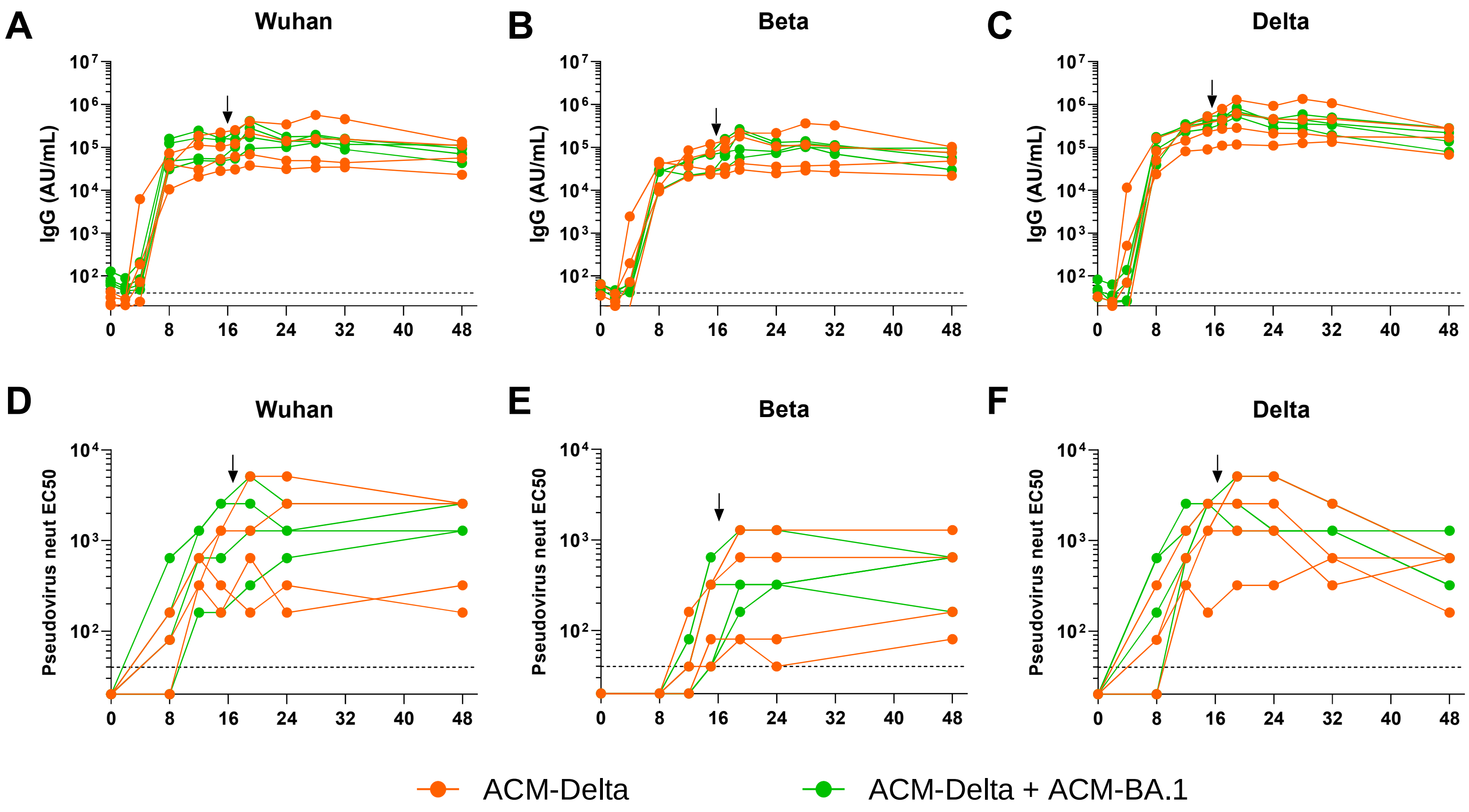

Supplemental Figure S2. Longitudinal antibody responses in PARVAX boosted and not boosted non-human primates. **A-C)** Longitudinal Wuhan (A), Beta (B) and Delta (C) (D) binding antibody (AU/mL) responses up to week 48 after prime (ACM-Delta) administration. **D-F)** Longitudinal Wuhan (D), Beta (E) and Delta (F) neutralizing antibody responses up to week 48 after prime administration. Arrows indicate timing of second PARVAX administration (ACM-BA.1 or Saline).

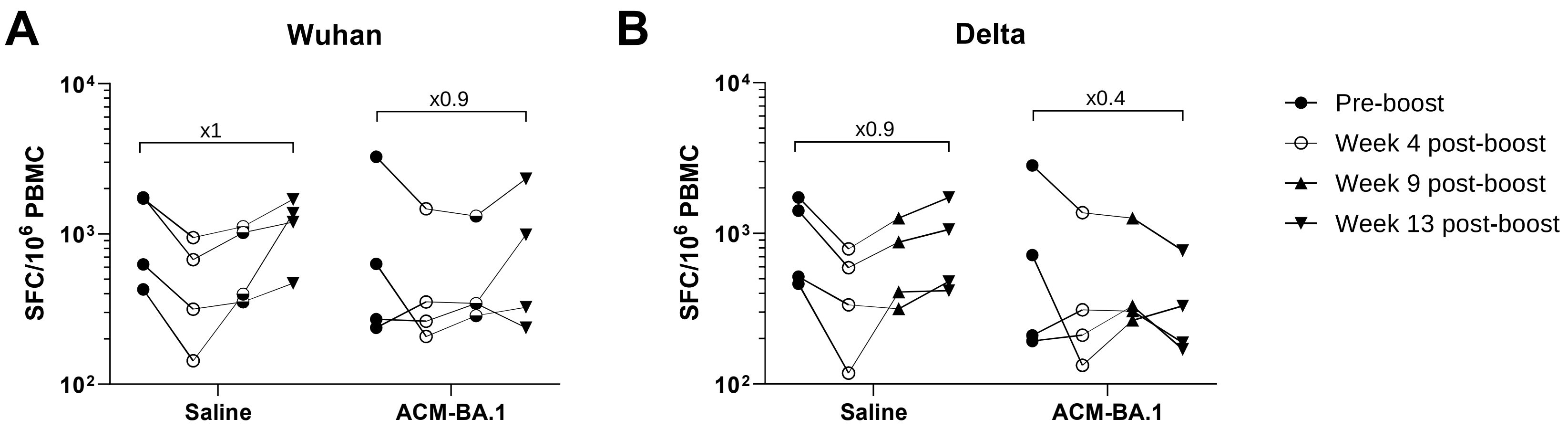

Supplemental Figure S3. Cellular immune responses in PARVAX boosted and not boosted non-human primates.. **A-B)** IFN-gamma spot forming cells (SFC) after Wuhan (A) or Delta (B) Spike protein peptide stimulation of peripheral blood mononuclear cells (PBMC) extracted on week 15 (pre-boost), week 19 (4 weeks post-boost), week 24 (9 weeks post-boost) and week 28 (13 weeks post-boost).
